# SUCCOR Risk: Design and Validation of a Recurrence Prediction Index for Early-Stage Cervical Cancer

**DOI:** 10.1245/s10434-022-11671-5

**Published:** 2022-04-16

**Authors:** Nabil Manzour, Luis Chiva, Enrique Chacón, Nerea Martin-Calvo, Felix Boria, José A. Minguez, Juan L. Alcazar, On behalf of the SUCCOR Study Group, Vanna Zanagnolo, Denis Querleu, Mihai Căpîlna, Anna Fagotti, Ali Kucukmetin, Constantijne Mom, Galina Chakalova, Shamistan Aliyev, Mario Malzoni, Fabrice Narducci, Octavio Arencibia, Francesci Raspagliesi, Tayfun Toptas, David Cibula, Dilyara Kaidarova, Mehmet Meydanli, Mariana Tavares, Dmytro Golub, Anna Perrone, Robert Poka, Dimitrios Tsolakidis, Goran Vujić, Marcin Jedryka, Petra Zusterzeel, Jogchum Beltman, Frédéric Goffin, Dimitros Haidopoulos, Herman Haller, Robert Jach, Iryna Yezhova, Igor Berlev, Margarida Bernardino, Rasiah Bharathan, Maximilian Lanner, Vladyslav Sukhin, Jean G. Feron, Robert Fruscio, Kersti Kukk, Jordi Ponce, Nabil Abdalla, Özgür Akbayir, Sedat Akgöl, Elif Aksahin, Shamistan Aliyev, Maria Alonso-Espias, Igor Aluloski, Claudia Andrade, Nikola Badzakov, Rosa Barrachina, Giorgio Bogani, Eduard-Aexandru Bonci, Hélène Bonsang-Kitzis, Cosima Brucker, Laura Cárdenas, Andrea Casajuana, Pere Cavalle, Jorge Cea, Benito Chiofalo, Gloria Cordeiro, Pluvio Coronado, Maria Cuadra, Javier Díez, Teresa Diniz da Costa, Santiago Domingo, Lukas Dostalek, Fuat Demirkiran, Diego Erasun, Mathias Fehr, Sergi Fernandez-Gonzalez, Soledad Fidalgo, Gabriel Fiol, Khadra Galaal, José García, Gerhard Gebauer, Fabio Ghezzi, Juan Gilabert, Nana Gomes, Elisabete Gonçalves, Virginia Gonzalez, Frederic Grandjean, Miriam Guijarro, Frédéric Guyon, Jolien Haesen, Gines Hernandez-Cortes, Sofía Herrero, Imre Pete, Ioannis Kalogiannidis, Erbil Karaman, Andreas Kavallaris, Lukasz Klasa, Ioannis Kotsopoulos, Stefan Kovachev, Meelis Leht, Arantxa Lekuona, Mathieu Luyckx, Michael Mallmann, Gemma Mancebo, Aljosa Mandic, Tiermes Marina, Victor Martin, María Belén Martín-Salamanca, Alejandra Martinez, Gesine Meili, Gustavo Mendinhos, Liliana Mereu, Milena Mitrovic, Sara Morales, Enrique Moratalla, Bibiana Morillas, Eva Myriokefalitaki, Maja PakižImre, Stamatios Petousis, Laurentiu Pirtea, Natalia Povolotskaya, Sonia Prader, Alfonso Quesada, Mikuláš Redecha, Fernando Roldan, Philip Rolland, Reeli Saaron, Cosmin-Paul Sarac, Jens-Peter Scharf, Špela Smrkolj, Rita Sousa, Artem Stepanyan, Vladimír Študent, Carmen Tauste, Hans Trum, Taner Turan, Manuela Undurraga, Arno Uppin, Alicia Vázquez, Ignace Vergote, George Vorgias, Ignacio Zapardiel

**Affiliations:** 1 Department of Gynecology, Clinica Universidad de Navarra, Pamplona, Spain; 2 Department of Preventive Medicine and Public Health, Universidad de Navarra, Pamplona, Spain; 3 Department of Gynecology, Clínica Universidad de Navarra, Madrid, Spain

## Abstract

**Objective:**

Based on the SUCCOR study database, our primary objective was to identify the independent clinical pathological variables associated with the risk of relapse in patients with stage IB1 cervical cancer who underwent a radical hysterectomy. Our secondary goal was to design and validate a risk predictive index (RPI) for classifying patients depending on the risk of recurrence.

**Methods:**

Overall, 1116 women were included from January 2013 to December 2014. We randomly divided our sample into two cohorts: discovery and validation cohorts. The test group was used to identify the independent variables associated with relapse, and with these variables, we designed our RPI. The index was applied to calculate a relapse risk score for each participant in the validation group.

**Results:**

A previous cone biopsy was the most significant independent variable that lowered the rate of relapse (odds ratio [OR] 0.31, 95% confidence interval [CI] 0.17–0.60). Additionally, patients with a tumor diameter >2 cm on preoperative imaging assessment (OR 2.15, 95% CI 1.33–3.5) and operated by the minimally invasive approach (OR 1.61, 95% CI 1.00–2.57) were more likely to have a recurrence. Based on these findings, patients in the validation cohort were classified according to the RPI of low, medium, or high risk of relapse, with rates of 3.4%, 9.8%, and 21.3% observed in each group, respectively. With a median follow-up of 58 months, the 5-year disease-free survival rates were 97.2% for the low-risk group, 88.0% for the medium-risk group, and 80.5% for the high-risk group (*p* < 0.001).

**Conclusion:**

Previous conization to radical hysterectomy was the most powerful protective variable of relapse. Our risk predictor index was validated to identify patients at risk of recurrence.

**Supplementary Information:**

The online version contains supplementary material available at 10.1245/s10434-022-11671-5.

Despite population screening and widespread use of a vaccine against it, cervical cancer is still one of the most common gynecological malignancies.^1^

For years, open and minimally invasive surgery (MIS), either by laparoscopy or robotics, were considered acceptable approaches for radical hysterectomy in patients with early-stage cervical cancer.^2–7^ However, publication of the LACC trial and SUCCOR study demonstrated higher relapse and mortality rates in patients who underwent MIS than those who underwent open surgery.^8,9^

After publication of the LACC trial,^8^ we observed a growing interest in understanding why patients who underwent radical hysterectomy by MIS for early cervical cancer presented a higher risk of relapse and mortality than others.

Updated information on the outcomes of patients who undergo a radical hysterectomy in Europe was missing. Therefore, we designed the SUCCOR study to compare the risks of relapse and overall survival (OS) in women with stage IB1 cervical cancer who underwent radical hysterectomy by MIS or open abdominal surgery between 2013 and 2014. The results of this study’s primary analyses showed that MIS was associated with a higher risk of relapse and death than open surgery.^9^

The primary goal of the SUCCOR risk study was to identify the variables that independently predict the risk of relapse in European patients with early cervical cancer after radical hysterectomy. As a secondary objective, we aimed to design a clinical prediction index that evaluates the risk of relapse based on the independent variables. Finally, we pursued to validate this prediction index’s efficacy.

## Methods

The SUCCOR study is a European, multicenter, retrospective cohort study with the primary goal of analyzing disease-free survival (DFS) and OS after radical hysterectomy in women with early-stage cervical cancer who underwent surgery in Europe in 2013 and 2014.

### Inclusion and Exclusion Criteria

Inclusion and exclusion criteria have been published by our group elsewhere.^10^

Unlike the original SUCCOR study, our study included patients with previous cone biopsy because we considered this may be a key variable to predict the risk of relapse.

### Accrual and Data Source

We invited all members of the European Society of Gynaecological Oncology (ESGO) to participate in this study. Researchers from 126 institutions belonging to 29 European countries registered and contributed to the project. After obtaining ethical consent from our central Institutional Review Board, we required a Certificate of Approval or a Letter of Exemption by the local Ethics Committees from all investigators.

An anonymized complete case record form including 123 items was sent to all investigators. After completing the case collection, all researchers signed a detailed final declaration affirming that all the submitted data entirely matched the data within patients’ charts. As far as each researcher was able to analyze, the data included all cases at the respective institutions.

### Statistical Analyses

We randomly divided our sample into testing and validation cohorts at a ratio of 60% versus 40%. We used Student’s *t*-test for quantitative variables and Pearson’s Chi-square test for qualitative variables to compare the two sets of main variables.

The testing cohort was used to identify clinical and pathological variables independently associated with the outcome and to define the predictive index score of the risk of relapse. Based on the existing evidence, we chose a list of variables and calculated the odds ratio (OR) and 95% confidence interval (CI) for the risk of relapse using simple logistic regression models. All variables with a *p*-value <0.20 in the univariate analyses were introduced in a forward stepwise procedure. Two significance levels were specified in the process: 5% for predictor addition to the model and 10% for predictor removal. *β* coefficients were divided by the smallest value and rounded to integers to calculate each variable’s ratio in the index. The area under the receiver operating characteristic (ROC) curve of the predictive index was calculated in both the testing and validation sets.

We applied the predictive index to calculate a score of the risk of relapse for each participant in the validation cohort and used simple logistic regression to estimate the predicted probability of relapse associated with the score as a quantitative variable. According to their risk of relapse, participants were classified into low (0–3 points), medium (4–6 points), or high (7–9 points) risk groups. We calculated the OR and 95% CI for the risk of relapse for each category using the lowest group as the reference. The linear trend across categories was also calculated.

We estimated the hazard ratio and 95% CI for DFS and OS for each risk group in the validation cohort, and performed statistical analyses using the SPSS 26.0 package (IBM Corporation, Armonk, NY, USA). All *p*-values are two-sided and statistical significance was defined as *p* < 0.05.

## Results

**Fig. 1:**
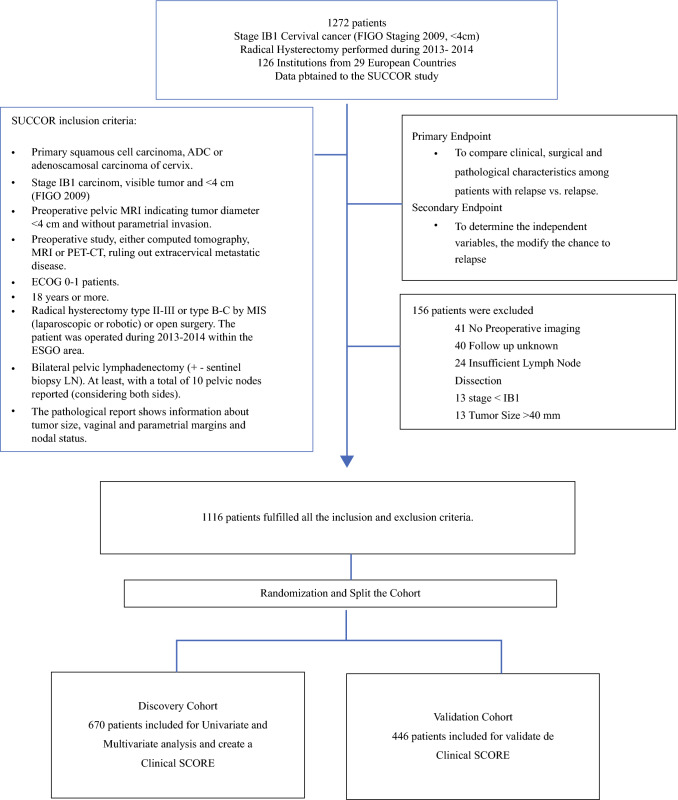
Study population. *FIGO* International Federation of Gynecology and Obstetrics, *MRI* magnetic resonance imaging, *PET* positron emission tomography, *CT* computed tomography, *ECOG* Eastern Cooperative Oncology Group, *MIS* minimally invasive surgery, *ESGO* European Society of Gynaecological Oncology, *LN* lymph node, *ADC* adenocarcinoma

From 15 May to 15 November 2019 we collected data from 1272 patients with stage IB1 cervical cancer (International Federation of Gynecology and Obstetrics [FIGO] 2009) who underwent radical hysterectomy in Europe between 2013 and 2014. Overall, 156 patients did not meet the inclusion criteria and were excluded from the study. Further analyses were performed with the remaining group of 1116 patients. We randomly divided the population into testing and validation cohorts, resulting in 670 and 446 patients in each, respectively. After a median follow-up of 58 months, we observed 81 (12.1%) and 45 (10.1%) relapse cases in the testing and validation sets, respectively (Fig. 1).

No differences in baseline characteristics were observed between the testing and validation groups, except for the surgical approach (*p* = 0.021) (Table 1).

**Table 1:** Selected baseline characteristics of patients with and without relapse after undergoing radical hysterectomy for stage IB1 cervical carcinoma in the discovery and validation cohorts [*N* = 1116]

Baseline characteristics	Discovery cohort	Validation cohort	*p*-Value
	[*n* = 670]	[*n* = 446]	
Age, years	47 (10.79)	46 (10.85)	0.08
BMI, kg/m^2^	25.40 (5.05)	25.41 (4.93)	0.97
*Heavy smokers (%)*			
No	373 (55.7)	249 (55.8)	0.99
Yes	132 (19.7)	88 (19.7)	
Not reported	165 (24.6)	109 (24.4)	
*Immunosuppression (%)*			
No	596 (89)	412 (92.4)	0.13
Yes	22 (3.3)	8 (1.8)	
Not reported	52 (7.8)	26 (5.8)	
*Preoperative clinical size (%)*			
<2 cm	374 (55.8)	275 (61.7)	0.51
>2 cm	283 (42.3)	168 (37.7)	
Not reported	13 (1.9)	3 (0.6)	
*Preoperative image size (%)*			
<2 cm	359 (53.6)	256 (57.4)	0.209
>2 cm	311 (46.4)	190 (42.6)	
*Cone biopsy before surgery (%)*			
No	423 (63.1)	271 (60.8)	
Yes	242 (36.1)	175 (39.2)	0.118
Not reported	5 (0.8)		
Radical hysterectomy report			
*Surgical approach (%)*			
Open	342 (51)	259 (58.1)	**0.021**
MIS	328 (49)	187 (41.9)	
*Type of RH [P-R/Q-M] (%)*			
Type II or B	194 (29.3)	127 (28.6)	0.494
Type III or C	449 (67.8)	309 (69.6)	
Not reported	19 (2.9)	8 (1.8)	
*First surgeon (%)*			
Fellow and junior surgeon	157 (23.4)	91 (20.4)	0.167
Senior surgeon	498 (74.3)	350 (78.5)	
Not reported	15 (2.2)	5 (1.1)	
Largest diameter in the pathology report, mm			
*Largest tumor diameter (%)*			
<2 cm	372 (55.5)	247 (55.4)	0.963
>2 cm	298 (44.5)	199 (44.6)	
*Final histology (%)*			
Squamous	463 (69.1)	297 (66.6)	0.456
Adenocarcinoma	188 (28.1)	131 (29.4)	
Adenosquamous	19 (2.8)	18 (4)	
*Final histological grade (%)*			
1	95 (14.2)	79 (17.7)	0.337
2	279 (41.6)	189 (42.4)	
3	196 (29.3)	116 (26)	
Not reported	100 (14.9)	62 (13.9)	
*LVSI(%)*			
No LVSI	361 (53.9)	250 (56.1)	0.496
Presence of LVSI	234 (34.9)	141 (31.6)	
Not reported	75 (11.2)	55 (12.3)	
*Depth of invasion (%)*			
Superficial <1/3	141 (21)	116 (26)	0.183
Intermediate >1/3 and <2/3	182 (27.2)	121 (27.1)	
Deep >2/3	172 (25.7)	96 (21.5)	
Not reported	175 (26.1)	113 (25.3)	
*Margins status (%)*			
Negative	616 (91.9)	415 (93)	0.732
Positive or close <2 mm	53 (7.9)	30 (6.7)	
Not reported	1 (0.1)	1 (0.2)	
*Lymph node status (%)*			
Negative	595 (88.8)	388 (87)	0.361
Positive	75 (11.2)	58 (13)	
*FIGO staging 2018 (%)*			
IB1	296(44.2)	191 (42.8)	0.713
IB2	283 (42.2)	187 (41.9)	
II–III <4 cm	90 (13.4)	66 (14.8)	
Not reported	1 (0.1)	2 (0.4)	
*Adjuvant therapy (%)*			
Without adjuvant therapy	363 (54.2)	255 (57.2)	0.518
With adjuvant therapy	304 (45.4)	188 (42.2)	
Not reported	3 (0.4)	3 (0.7)	
*Relapse (%)*			
No	589 (87.9)	401 (89.9)	0.301
Yes	81 (12.1)	45 (10.1)	

Counts in the weighted cohort may not sum to the expected totals due to rounding, and percentages may not total 100 due to rounding. Disagreements between numbers and percentages in the weighted cohort are the result of rounding of non-integer number values. Distributions of categorical variables were compared using the Chi-square test in the unweighted cohort, and quantitative variables were compared using Student’s *t*-test in the unweighted cohort

*BMI* body mass index, *MIS* minimally invasive surgery, *RH* radical hysterectomy, *P-R* Piver–Rutledge classification, *Q-M* Querleu-Morrow, *LVSI* lymphovascular space invasion, *FIGO* International Federation of Gynecology and Obstetrics

### Univariate Analysis

In the testing cohort, we observed that preoperative cone biopsy was inversely associated with the risk of relapse (OR 0.31, 95% CI 0.17–0.60; *p* < 0.001). Furthermore, we found that women who relapsed were more likely to have large tumors (>2 cm) on the preoperative imaging assessment (OR 2.15, 95% CI 1.33–3.50; *p* = 0.002) and underwent MIS more frequently than those without relapses (OR 1.61, 95% CI 1.00–2.57; *p* = 0.049). Regarding pathological findings, we observed that women who relapsed had higher proportions of large tumors (OR 1.97, 95% CI 1.23–3.16; *p* = 0.005), deeper stromal invasion (OR 2.31, 95% CI 1.14–4.67; *p* = 0.020), a higher rate of positive or close margins (OR 2.04, 95% CI 1.01–4.15; *p* = 0.048), and a higher FIGO 2018 pathological stage (OR 2.14, 95% CI 1.29–3.57; *p* = 0.003) (Table 2).

**Table 2:** Univariable analysis of the discovery cohort with cervical cancer

	OR (95% CI)	*p*-Value
*Preoperative clinical size*		
<2 cm	1 (Reference)	0.561
>2 cm	1.083 (0.828–1.417)	
*Preoperative image size*		
<2 cm	1 (Reference)	**0.002**
>2 cm	2.151 (1.332–3.474)	
*Cone biopsy before surgery*		
No	1 (Reference)	**<0.001**
Yes	0.307 (0.166–0.596)	
*Surgical approach*		
Open	1 (Reference)	**0.049**
MIS	1.605 (1.001–2.573)	
*First surgeon*		
Fellow and junior surgeon	1 (Reference)	0.818
Senior surgeon	0.938 (0.546–1.612)	
*Largest tumor diameter*		
<2 cm	1 (Reference)	**0.005**
>2 cm	1.972 (1.230–3.163)	
*Final histological grade*		
1	1 (Reference)	0.096
2–3	1.993 (0.884–4.493)	
*LVSI*		
No LVSI	1 (Reference)	0.470
Presence of LVSI	1.393 (0.567–3.419)	
*Depth of invasion*		
Superficial <1/3	1 (Reference)	**0.020**
Intermediate or deep >1/3	2.307 (1.138–4.674)	
*Margins status*		
Negative	1 (Reference)	**0.048**
Positive or close <2 mm	2.043 (1.005–4.151)	
*Lymph node status*		
Negative	1 (Reference)	0.469
Positive	1.289 (0.649–2.561)	
*FIGO staging 2018*		
IB1	1 (Reference)	**0.003**
IB2–II–III <4 cm	2.141 (1.285–3.567)	
*Adjuvant therapy*		
Without adjuvant therapy	1 (Reference)	0.984
With adjuvant therapy	1.005 (0.630–1.602)	

Based on the existing evidence, a list of variables were chosen and the OR and 95% CI were calculated for the risk of relapse using simple logistic regression models to establish clinical and pathological variables independently associated with the outcome

*OR* odds ratio, *CI* confidence interval, *MIS* minimally invasive surgery, *LVSI* lymphovascular space invasion, *FIGO* International Federation of Gynecology and Obstetrics

### Multivariate Analysis and Development of a Clinical Risk Score

Stepwise regression in the testing cohort resulted in an intercept of −3 and the following *β* regression coefficients: 1.04 for the absence of previous cone biopsy, 0.70 for the MIS approach, and 0.56 for tumor size >2 cm on the preoperative imaging assessment.

As explained in the Methods, we modified the calculated coefficients to obtain the following formula to predict the risk index:

Score = 4 (no cone biopsy) + 3 (MIS approach) + 2 (tumor size >2 cm on imaging) (Table 3).

**Table 3:** Index risk variables

	B	*p*-Value
Constant	−3.441	
Cone biopsy before surgery	1.040	0.002
Approach	0.699	0.005
Preoperative image size	0.564	0.036
Score = 4 (no cone biopsy) + 3 (MIS approach) + 2 (tumor size >2 cm on imaging)		

Variables were selected by stepwise analysis in the testing cohort to develop our algorithm to determine the risk of relapse, and the final score was obtained

*MIS* minimally invasive surgery

**Fig. 2:**
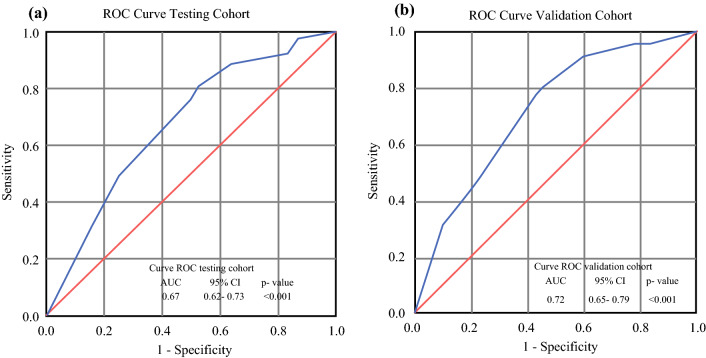
ROC curve with an AUC and 95% CIs for the **a** testing cohort and **b** validation cohort for the risk of relapse. *ROC* receiver operating characteristic, *AUC* area under the curve, *CIs* confidence intervals

We calculated a score for each participant in both the testing and validation cohorts using this formula. The area under the ROC curve in both the testing and validation groups is shown in Fig. 2 (*p* < 0.001).

**Fig. 3:**
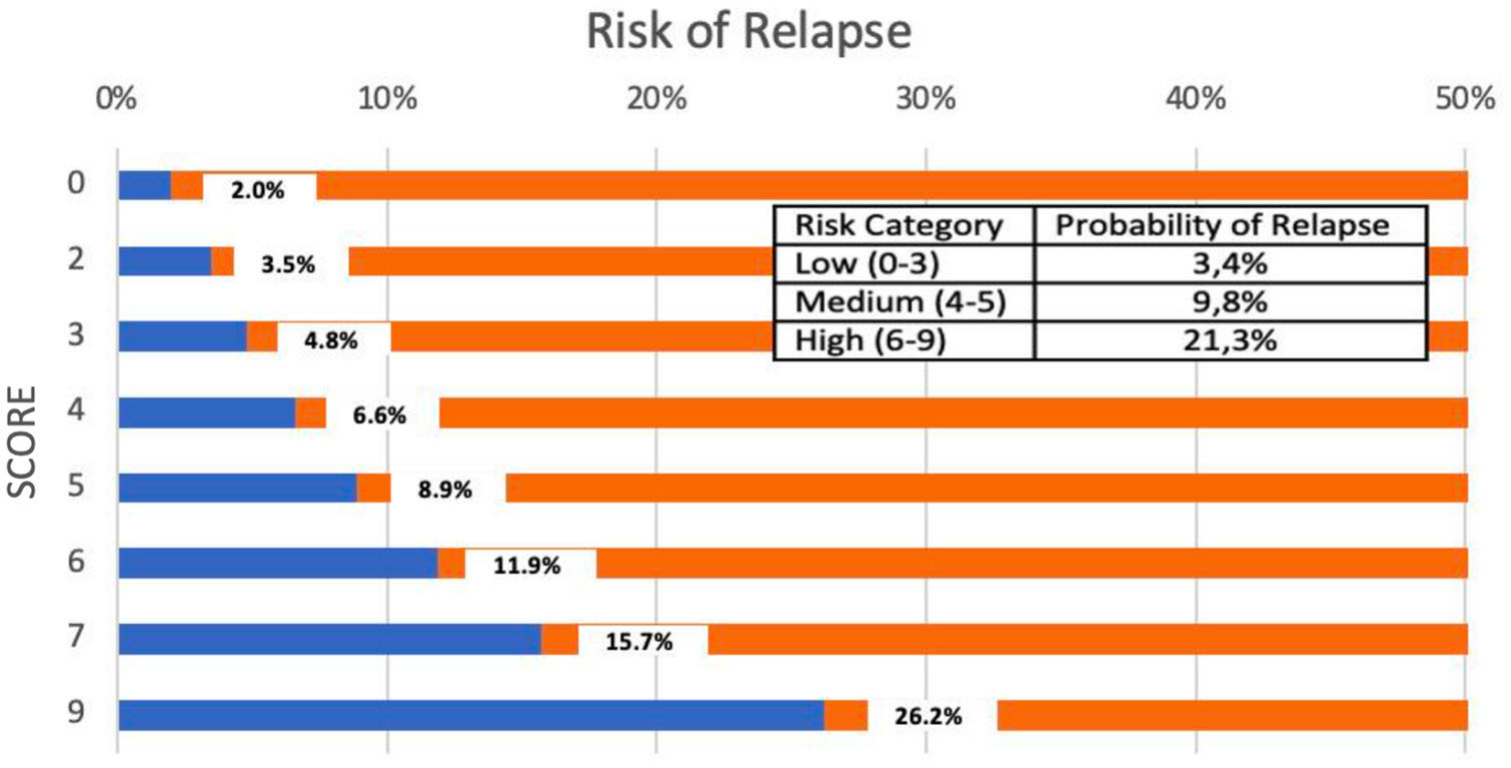
Predicted risk of relapse by score and risk group

Patients in the validation cohort were classified, according to their risk of relapse, into low (0–3 points), medium (4–6 points), or high (7–9 points) risk groups. The observed rates of relapse in each group were 3.4%, 9.8%, and 21.3%, respectively. The predicted developing risk of relapse by score and risk group is presented in Fig. 3.

We observed a significant linear association between the calculated score and the risk of relapse. Specifically, each extra point in the index was associated with a relative 38.1% increase in the relapse risk (*p* < 0.001). Moreover, we observed a 5.35-fold (95% CI 1.80–15.94; *p* = 0.003) and 9.80-fold (95% CI 3.25–29.67; *p* < 0.001) higher risk of relapse for women in the medium and high categories of the risk index than the risk for those in the lowest category. Additionally, we observed a significant linear trend across categories (*p* < 0.001) (electronic supplementary Fig. 1).

### Positive Predictive Value

The index predicting the likelihood of relapse for medium-risk women was 15% and for high-risk patients was 22%, assuming an overall relapse rate of 10% in the validation group.

### Disease-Free Survival and Overall Survival

**Fig. 4:**
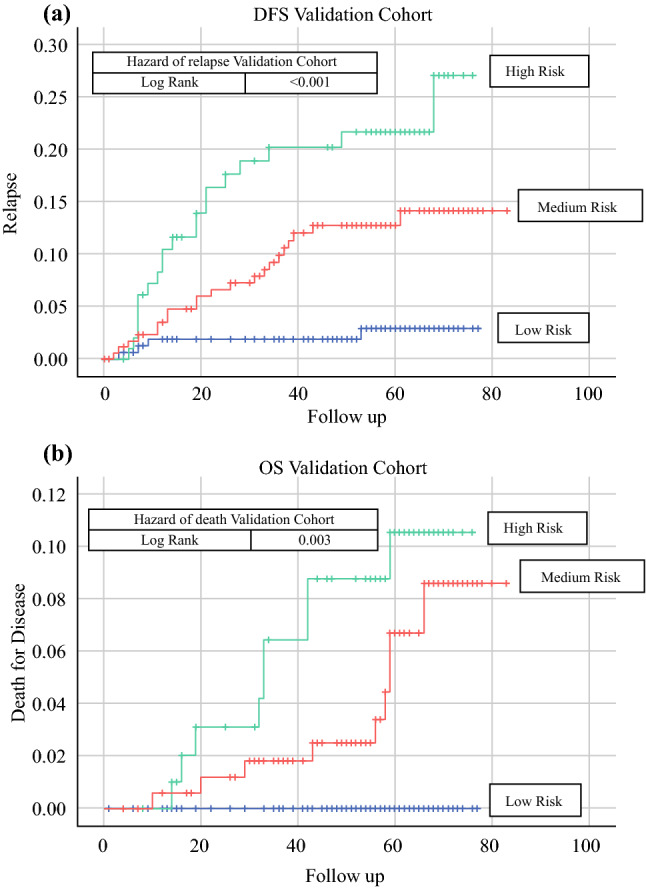
Hazard ratios for the **a** risk of relapse and **b** overall survival, by risk groups in the validation cohort (low, medium, and high risk). *OS* overall survival

The median follow-up of our population was 58 months. Four of 165 (2.4%) patients in the low-risk group, 21 of 179 (11.7%) patients in the medium-risk group, and 20 of 102 (19.6%) patients in the high-risk group suffered a relapse. The 5-year DFS rates were 97.2%, 88.0%, and 80.5% for the low-risk, medium-risk, and high-risk groups, respectively (log-rank *p* < 0.001) (Fig. 4a).

In terms of disease-related mortality in the validation cohort, we observed 0 of 163 (0%) patients in the low-risk group, 9 of 175 (5.1%) patients in the medium-risk group, and 9 of 101 (8.9%) patients in the high-risk group. The 5-year OS rates were 100%, 93.5%, and 90.0% for the low-risk, medium-risk, and high-risk groups, respectively (log-rank *p* = 0.003) (Fig. 4b).

### Subgroup Analysis Based on Adjuvant Treatment

#### Patients in the Validation Cohort without Adjuvant Treatment.

With each participant’s score, we created the area under the curve shown in electronic supplementary Fig. 2a. Three of 123 (2.4%) patients in the low-risk group, 6 of 77 (7.8%) patients in the medium-risk group, and 10 of 55 (18.2%) patients in the high-risk group suffered a relapse. The 5-year DFS rates were 97.1%, 92.0%, and 83.2% for the low-risk, medium-risk, and high-risk groups, respectively (log-rank *p* = 0.002) (electronic supplementary Fig. 3a).

In terms of disease-related mortality in patients without adjuvant treatment, we observed 0 of 122 (0%) patients in the low-risk group, 1 of 76 (1.3%) patients in the medium-risk group, and 4 of 55 (7.3%) patients in the high-risk group. The 5-year OS rates were 100%, 98.6.0%, and 92.3% for the low-risk, medium-risk, and high-risk groups, respectively (log-rank *p* = 0.006) (electronic supplementary Fig. 3b).

#### Patients in the Validation Cohort with Adjuvant Treatment

Again, using each score, we constructed the area under the curve shown in electronic supplementary Fig. 2b. One of 42 (2.4%) patients in the low-risk group, 15 of 99 (15.2%) patients in the medium-risk group, and 10 of 47 (21.3%) patients in the high-risk group relapsed. The 5-year DFS rates were 97.4%, 84.5%, and 77.4% for the low-risk, medium-risk, and high-risk groups, respectively (log-rank *p* = 0.039) (electronic supplementary Fig. 3c).

Regarding disease-related mortality in patients with adjuvant treatment, we observed 0 of 41 (0%) patients in the low-risk group, 8 of 96 (8.3%) patients in the intermediate-risk group, and 5 of 46 (10.9%) patients in the high-risk group. The 5-year OS rates were 100%, 88.7%, and 87.0% for the low-risk, intermediate-risk, and high-risk groups, respectively (log-rank *p* = 0.151) (electronic supplementary Fig. 3d).

## Discussion

In 2018, the LACC trial published by Ramirez et al. completely changed the perspective of the surgical approach in early cervical cancer. For the first time, a randomized clinical trial demonstrated the potential harm of minimally invasive surgery in patients who underwent radical hysterectomy.^8^ Our study, SUCCOR Risk, has the main objective of identifying the independent variables that predict the risk of relapse in patients with cervical cancer smaller than 4 cm after radical hysterectomy in a large European population.

Our study included 1116 European women with stage IB1 cervical cancer (FIGO 2009) who underwent radical hysterectomy between 2013 and 2014, with strict inclusion/exclusion criteria and an extended follow-up. In the multivariate analysis, we found three significant independent variables that modify the risk of relapse: a previous cone biopsy, the type of surgical approach, and the tumor diameter on preoperative imaging that independently predicted the risk of relapse. Furthermore, these variables predicted the risk of recurrence in our population better than the classic pathological variables.

The most relevant finding of this study was the powerful impact of the previous cone biopsy to predict the risk of relapse. Moreover, cone biopsy has greater relevance than surgical approach or tumor size in our population. Although, in our database, the preoperative cone biopsy was associated with smaller cervical cancer tumors (*p* < 0.001) (electronic supplementary Table 1), interestingly, when we analyzed the relationship between cone biopsy and the likelihood of relapse, we observed that the cone has a similar grade of protective effect in smaller tumors ≤2 cm than in tumors >2 cm (OR 0.25, 95% CI 0.13–0.48, *p* < 0.001; and OR 0.27, 95% CI 0.10–0.76, *p* = 0.013, respectively) (electronic supplementary Table 2). In fact, in a recent article published by our group, we demonstrated that patients with previous cone biopsy operated by MIS have the same outcomes as patients operated by open surgery,^10^ which is consistent with previous evidence.^11,12^

In another recent study that evaluated relapse risk factors on early cervical cancer after surgery, Cibula et al.^13^ did not include the previous conization as a potential risk factor, and, logically, it was not evaluated in the univariate and multivariate analyses. Similarly, the LACC trial and subsequent publications discovered the negative impact of the surgical approach (MIS);^8,14^ however, no publications considered conization as a potential protective maneuver.

Surgical approach was the second most influential risk factor for relapse in the multivariate analysis. Again, using data from the SUCCOR cohort, our group published the negative impact of MIS, with similar results, excluding conization, and using thorough statistical strategies to control for confounding factors.^9^

Interestingly, cone biopsy and the type of surgical approach are modifiable factors to consider when operating patients with cervical cancer. Never before has a cone biopsy been considered under this perspective in the literature. The best explanation for this remarkable finding is the potential role of cone biopsy in avoiding tumor spread during radical hysterectomy.

Finally, the tumor diameter evaluated by preoperative imaging (≤2 cm vs. >2 cm) was the third independent factor predicting the risk of relapse. However, this assessment was not considered part of the standard work-up until publication of the new 2018 FIGO staging. Before then, clinical visual inspection was the primary tool to measure and stage cervical tumors. However, the ESGO Guidelines of Quality indicators for surgical treatment of cervical cancer now consider pelvic magnetic resonance imaging (MRI) or expert vaginal ultrasound as required examinations.^15^

Early cervical cancer treatment efforts have focused on understanding the clinical and pathological variables that might predict potential relapses to allow clinicians to indicate adjuvant therapy when a high risk of recurrence is suspected.^4,16,17^ Pathological findings in radical hysterectomy specimens, such as tumor diameter, depth of invasion, lymphovascular space invasion, margin status, or nodal metastases, are considered risk factors for relapse.^18–28^ Interestingly, after running a logistic regression forward stepwise procedure, no pathological findings remained in our study and only clinical variables remained in the multivariate analysis.

We attempt to explain these surprising results by highlighting that our population received a high rate of adjuvant therapy (44%). This might explain why we did not observe any of the variables classically described as independent risk factors for relapse. Moreover, the high rate of adjuvant treatment allows unexpected but useful variables to emerge in our study.

Assuming the forenamed independent variables, we designed a risk-predicting score that accurately discriminates the risk of relapse in our population. To define the risk of relapse, we outlined three discriminating risk groups. Finally, we verified this prediction index in a separate validation cohort.

Our risk-predicting index, using the variables independently associated with relapse risk, showed a discrimination capacity of 67% in the testing set and 71% in the validation test. This means that taking two women at random, one who will relapse and one who will not, the possibility that the index will correctly classify them is approximately 70%, which is a moderate but acceptable power to discriminate and classify a patient with risk of relapse.

The positive predictive value (PPV) of the index indicates the possibility of relapse of a female for whom the predictive index has classified as positive (more than 3 or 6 points depending on the cut-off). Since the PPV depends on the outcome’s prevalence, the same index may present different PPV values in diverse populations. To be conservative, we introduced the PPV of the index for a relapse rate of 10%, but its value would be greater in groups with higher rates of relapse.

When analyzing the effectiveness of the risk predictor index according to having received adjuvant treatment or not, we confirmed the effectiveness and usefulness of our score in patients who have not received adjuvant therapy, with a predictive capacity similar to that of the total validation population. In contrast, we observed that in patients who have received adjuvant treatment, the ability to correctly classify patients according to their risk of death by disease disappears. The use of adjuvant therapies is a factor that may modify the efficacy of the logistic models, altering the predictive power. These findings may result from the adjuvant treatment’s transformation of the natural disease course.

Our study was an observational retrospective project and therefore has intrinsic limitations. However, observational studies can contribute valuable evidence, suggesting causal associations when designed and conducted using rigorous methods. Due to the retrospective design of our study, the possibility of presenting bias must be considered. Nevertheless, the accuracy of the data relies on extraction of the data from medical records following a thorough protocol. Furthermore, despite the strict inclusion and exclusion criteria control, the sample’s variability resulted in wide CIs. Therefore, we acknowledge that the observed estimates may represent the upper bound of the natural association between the predictive score and the relapse risk. Finally, the use of strict inclusion and exclusion criteria may reduce the extent to which the research findings can be applied to settings other than those in which the initial tests were performed.

Overall, the strengths of this study included an extraordinary collaborative effort of collecting comprehensive data of 1272 patients between 126 European institutions from 29 countries. The final cohort, including 1116 patients who underwent radical hysterectomy for stage IB1 cervical cancer between 2013 and 2014, was intensely analyzed. As a result, our project gathered one of the most extensive populations of patients with stage IB1 cervical cancer undergoing radical hysterectomies ever collected in Europe over a 2-year period. In addition, we designed a strict list of inclusion and exclusion criteria, which allowed for better control of confounding factors. Thus, we believe that our findings may not be attributable to confounding factors and instead represent a real biological effect. Moreover, we calculated the sensitivity and specificity of the index so that the PPV can be calculated for different populations with different relapse rates.

## Conclusions

In summary, we found a set of variables that conjunctly offer a new perspective for predicting disease relapse in this thoroughly analyzed European population with early cervical cancer. Overall, previous cone biopsy is a predictable protective variable to be considered for future research. Our index may be a complementary tool to the risk classification and classic risk factors. Its purpose is to modulate therapeutic decision making, especially in those intermediate-risk patients or those where it is not clear if the best therapeutic attitude is the close follow-up or adjuvant treatment.

## Supplementary Information

Below is the link to the electronic supplementary material.

Supplementary FIG. 1 Odds ratio plot, for three groups of risk. The low-risk group was taken as a reference to compare the increase in relapse risk with the medium- and high-risk groups (JPG 109kb)

Supplementary FIG. 2 ROC curve with an AUC and 95% CIs for validation cohort patients (a) without adjuvant treatment and (b) with adjuvant treatment, for the risk of relapse. ROC receiver operating characteristic, AUC area under the curve, CI confidence interval (PNG 563 kb)

Supplementary FIG. 3 Hazard ratios for the (a) risk of relapse and (b) overall survival, by risk groups in the validation cohort without adjuvant treatment (low, medium, and high risk), and (c) risk of relapse and (d) overall survival, by risk groups in the validation cohort with adjuvant treatment (low, medium, and high risk) (PNG 595 kb)

Supplementary file4 (DOCX 13 kb)

Supplementary file5 (DOCX 13 kb)
