# Supplementary figures and images for: SUCCOR Risk: Design and Validation of a Recurrence Prediction Index for Early-Stage Cervical Cancer
Source: Ann Surg Oncol. 2022 Apr 16;29(8):4819–29. doi: 10.1245/s10434-022-11671-5 (PMC9246807; doi:10.1245/s10434-022-11671-5)

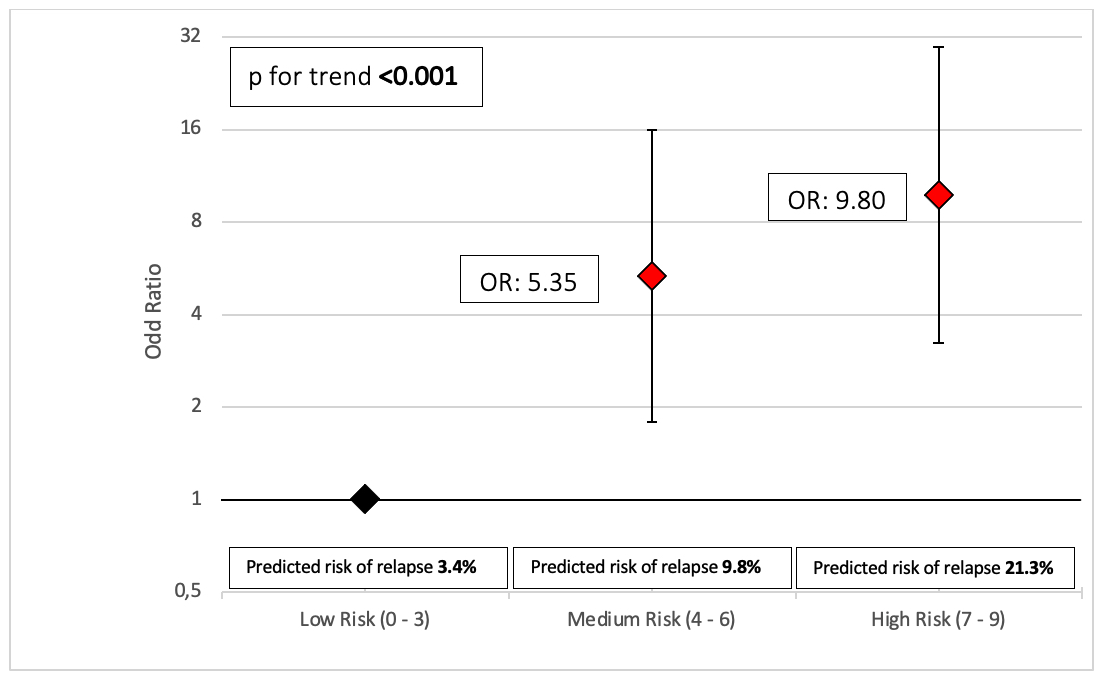

Supplement: Supplementary file 1 — Supplementary FIG. 1 Odds ratio plot, for three groups of risk. The low-risk group was taken as a reference to compare the increase in relapse risk with the medium- and high-risk groups (JPG 109kb) [file 10434_2022_11671_MOESM1_ESM.jpg]

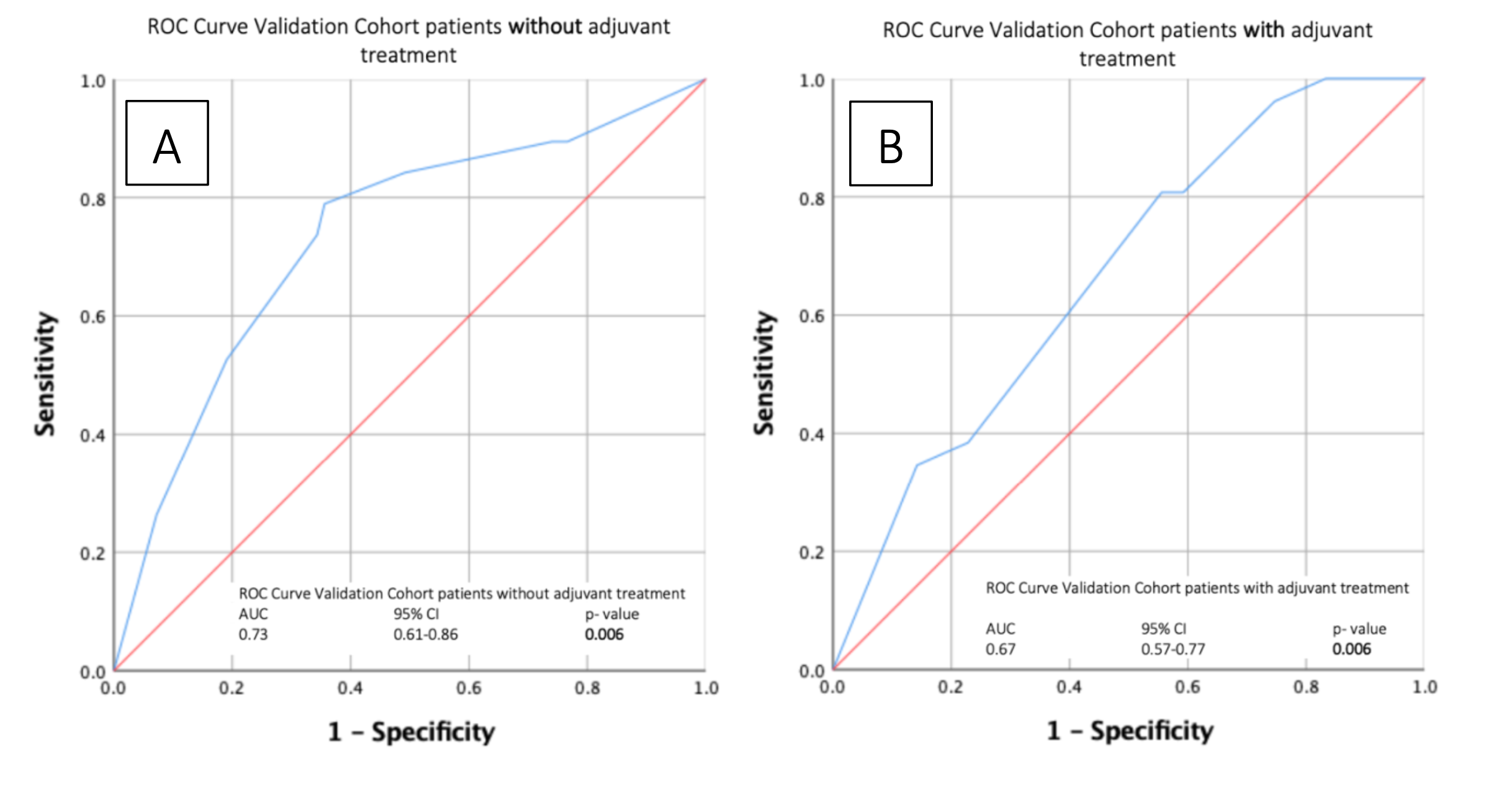

Supplement: Supplementary file 2 — Supplementary FIG. 2 ROC curve with an AUC and 95% CIs for validation cohort patients (a) without adjuvant treatment and (b) with adjuvant treatment, for the risk of relapse. ROC receiver operating characteristic, AUC area under the curve, CI confidence interval (PNG 563 kb) [file 10434_2022_11671_MOESM2_ESM.png]

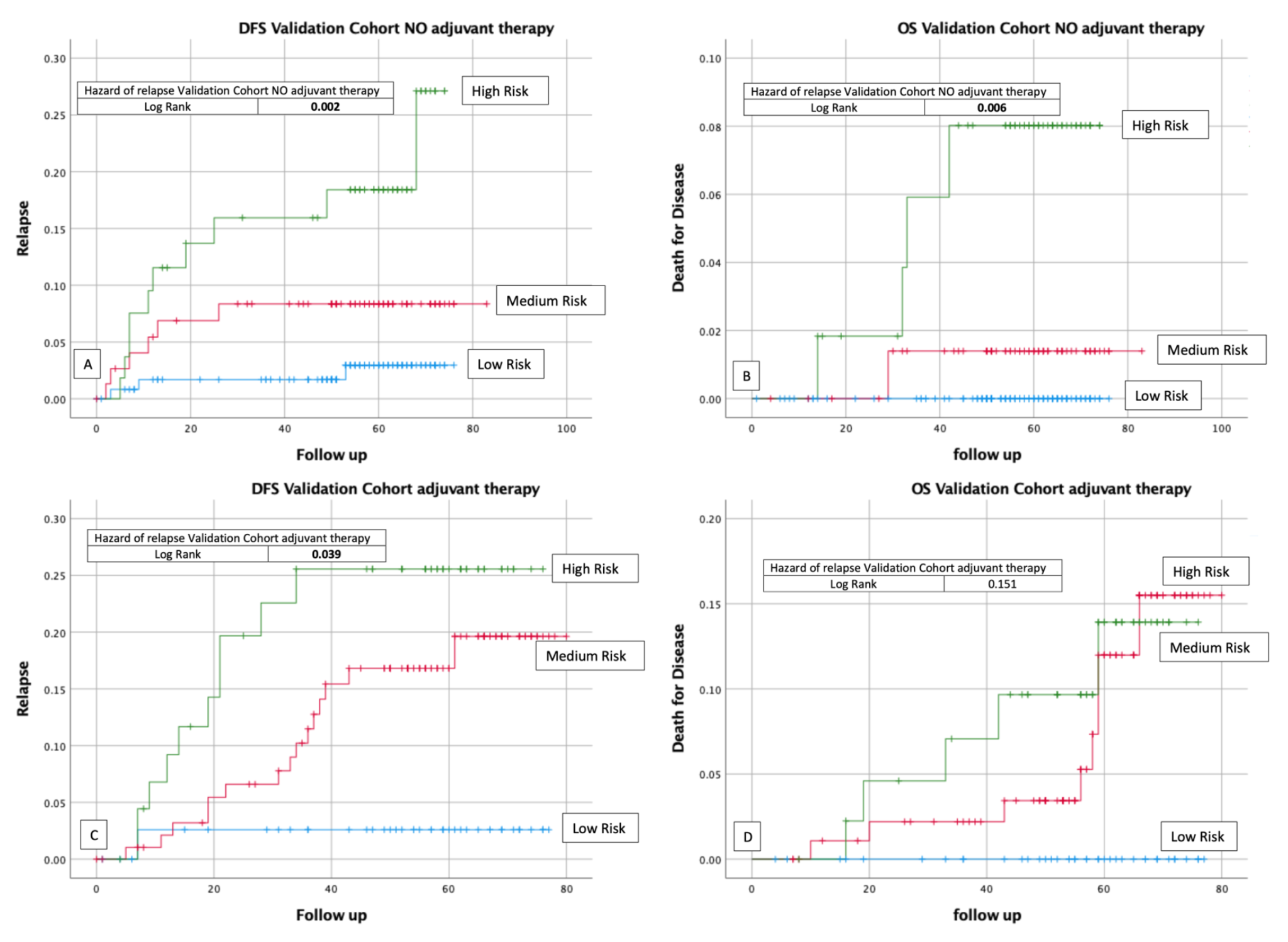

Supplement: Supplementary file 3 — Supplementary FIG. 3 Hazard ratios for the (a) risk of relapse and (b) overall survival, by risk groups in the validation cohort without adjuvant treatment (low, medium, and high risk), and (c) risk of relapse and (d) overall survival, by risk groups in the validation cohort with adjuvant treatment (low, medium, and high risk) (PNG 595 kb) [file 10434_2022_11671_MOESM3_ESM.png]
