# Supplementary material for: SUCCOR Risk: Design and Validation of a Recurrence Prediction Index for Early-Stage Cervical Cancer
Source: Ann Surg Oncol. 2022 Apr 16;29(8):4819–29. doi: 10.1245/s10434-022-11671-5 (PMC9246807; doi:10.1245/s10434-022-11671-5)
Supplement: Supplementary file 4 — Supplementary file4 (DOCX 13 kb) [file 10434_2022_11671_MOESM4_ESM.docx]

| **Table Supplementary 1.** Cone biopsy according to tumor size | | | |
| --- | --- | --- | --- |
| All patients N= 1116 | | | |
|  | **Tumors ≤2cm** | **Tumors >2cm** | p-value |
| **Cone Biopsy before surgery** |  |  |  |
| No | 325 (50.1) | 366 (82.1) | **<0.001** |
| Yes | 324 (49.9) | 80 (17.9) |  |
| Not reported |  | 5 (1.1) |  |

Table Supplementary 1. Analysis of cone biopsy performance prior to radical hysterectomy in patients with cervical cancer stage IB1 FIGO 2009 according to clinical preparative tumor size. Counts in the weighted cohort may not sum to expected totals owing to rounding. Percentages may not total 100 because of rounding, and disagreements between numbers and percentages in the weighted cohort are the result of rounding of non-integer number values. Distributions of categorical variables were compared using the chi-square test in the unweighted.
