# Supplementary material for: SUCCOR Risk: Design and Validation of a Recurrence Prediction Index for Early-Stage Cervical Cancer
Source: Ann Surg Oncol. 2022 Apr 16;29(8):4819–29. doi: 10.1245/s10434-022-11671-5 (PMC9246807; doi:10.1245/s10434-022-11671-5)
Supplement: Supplementary file 5 — Supplementary file5 (DOCX 13 kb) [file 10434_2022_11671_MOESM5_ESM.docx]

| **Table** **Supplementary 2.** Effect of cone biopsy on the risk of relapse. | | |
| --- | --- | --- |
| All patients N= 1116 | | |
|  | **Odd Ratio (CI 95%)** | **p-value** |
| **Cone Biopsy before surgery Tumors ≤2cm** |  |  |
| No | 1 (Reference) | **<0.001** |
| Yes | 0.254 (0.134- 0.479) |  |
| **Cone Biopsy before surgery Tumors >2cm** |  |  |
| No | 1 (Reference) | **0.013** |
| Yes | 0.268 (0.095- 0.762) |  |

Table Supplementary 2. Based on tumor size, the odds ratio (OR) and 95% CI for the risk of relapse were calculated using simple logistic regression models to establish the relationship between preoperative cone biopsy and tumor relapse.
